# Development and Characterization of Bio-Composites from the Plant Wastes of *Water Hyacinth* and Sugarcane Bagasse: Effect of Water Repellent and Gamma Radiation

**DOI:** 10.3390/polym15071609

**Published:** 2023-03-23

**Authors:** K. Z. M. Abdul Motaleb, Brigita Abakevičienė, Rimvydas Milašius

**Affiliations:** 1 Faculty of Mechanical Engineering and Design, Kaunas University of Technology, Studentų St. 56, 51424 Kaunas, Lithuania; 2 Department of Physics, Kaunas University of Technology, Studentų St. 50, 51368 Kaunas, Lithuania

**Keywords:** water hyacinth, sugarcane bagasse, plant waste, nonwoven reinforced composites, water repellent, gamma radiation

## Abstract

Plant waste is a huge source of natural fibers and has great potential in the field of reinforced polymer composites to replace the environmentally harmful synthetic composites. In this study, fibers were extracted from water hyacinth (WH) petiole and sugarcane bagasse (SB) to make nonwovens by wet-laid web formation, and reinforced on the polyester (P) and epoxy (E) resins to make four types of composites namely, water hyacinth nonwoven reinforced epoxy (WH + E), water hyacinth nonwoven reinforced polyester (WH + P), sugarcane bagasse nonwoven reinforced epoxy (SB + E) and sugarcane bagasse nonwoven reinforced polyester (SB + P) composites. Water repellent (WR) on the nonwovens and gamma radiation (GR) on the composites were applied to improve the hydrophobicity and mechanical properties, such as tensile strength (TS), elongation at break and tensile modulus (TM) of the composites. The morphological structure of the fiber surfaces and tensile fractures were analyzed by SEM. FTIR spectra showed changes in functional groups before and after treatment. XRD analysis exhibited an increase in crystallinity for gamma-irradiated composites and a decrease in crystallinity for WR-treated composites compared to untreated composites. The SB composites (SB + E, SB + P) and polyester composites (WH + P, SB + P) showed higher water absorbency and lower mechanical properties than the WH composites (WH + E, WH + P) and epoxy composites (WH + E, SB + E), respectively. Hydrophobicity improved significantly by approximately 57% (average) at a concentration of 10% WR. However, TS and TM were reduced by approximately 24% at the same concentration. Thus, 5% WR is considered an optimum concentration due to the very low deterioration of TS and TM (<10%) but significant improvement in hydrophobicity (~39%) at this dose. On the other hand, GR treatment significantly improved TS, TM and hydrophobicity by 41, 32 and 25%, respectively, and decreased Eb% by 11% at a dose of 200 krd. However, mechanical properties and hydrophobicity deteriorated with further increase in dose at 300 krd. Thus, 200 krd is considered the optimum dose of GR.

## 1. Introduction

In recent years, significant attention has been paid to environmentally friendly processes, sustainable materials and waste utilization, not only in research communities but also in industries to avoid the adverse environmental impacts caused by synthetic and non-biodegradable products [1,2]. For the last few decades, synthetic-fiber-reinforced polymer composites are widely used in various sectors due to their good mechanical properties and limitations of alternative solutions. However, these synthetic materials are environmentally harmful, responsible for global warming, toxic, carcinogenic, non-biodegradable, poorly recyclable, nonrenewable and consume a lot of energy [3]. Therefore, to ensure safe living, sustainable manufacturing and to solve the global energy crisis, the search for natural raw materials from renewable sources has become a pressing issue today [4]. In this context, natural fibers have great potential to be used as reinforcement in composite material to replace synthetic composites [5]. Natural-fiber-reinforced polymer composites (NFPCs) have numerous advantages such as low raw material and processing cost, light weight, high strength/weight ratio, biodegradable, renewable, recyclable, low processing energy, high specific strength and stiffness, thermal, electrical, and sound insulation properties, carbon neutrality and low pollutant emission [1,2,3,4,5,6,7,8]. 

In this investigation, fibers were extracted from the plant wastes of sugarcane bagasse and water hyacinth petiole to make different types of bio-composites. Sugarcane (*Saccharum officinarum*) plant is cultivated primarily to produce sugar by extracting sap from its stalks [9]. After the juice is extracted, the sapless stalk is known as bagasse and is mostly treated as waste. However, this bagasse contains a large number of fibers with a chemical composition of 46% cellulose, 24.5% hemicellulose, 19.5% lignin, 3.5% fat, 2.4% ash, and 2% silica [10]. On the other hand, water hyacinth (*Eichhornia crassipes*) is an aquatic plant that is grown naturally in a freshwater reservoir. It is often considered a problematic weed due to some dangerous effects such as covering the water body, reducing the oxygen level of the water, creating a risk to fish life, breeding mosquitoes, blocking the water flow of the river, disrupting the irrigation process, etc. [11,12,13,14]. However, the water hyacinth petiole contains a large number of cellulosic fibers with a chemical composition of 60% cellulose, 8% hemicellulose and 17% lignin [15]. Therefore, this problematic weed has great potential for use in composite reinforcement. 

The use of sugarcane bagasse fibers (SB) and water hyacinth fiber (WH) as a reinforcement in composite materials has already been introduced in several studies [15,16,17,18,19,20]. Through all of these studies, the WH and SB fibers have been used in a chopped/short form and reinforced sporadically. No study has been performed in which fibers were prefabricated in woven or nonwoven form. However, fiber orientation, amount, distribution, shape and adhesion to the matrix play an important role in composite performance [21]. The scattered reinforcement of fibers can lead to an uneven distribution and result in uneven mechanical properties at different places of the composites. Therefore, prefabrication to a nonwoven fabric from the chopped fibers can be a good option for a homogeneous distribution of the fibers and an easy reinforcement process. This study introduces an economic solution called wet-laid web formation in which the nonwovens were prepared from fiber pulp while immersed in the water. 

NFPCs have plenty of advantages as mentioned above; however, they contain some negative issues which cannot be ignored. One of the major disadvantages of NFPC is the high water absorbency, which results in poor adhesion to the hydrophobic polymer matrix and lower mechanical properties than synthetic composites. To overcome these problems, in this study, chemical treatment such as alkali and water repellent treatment and physical treatment, such as gamma radiation, was applied. The fibers were extracted by alkali treatment as well. The properties of fibers, as well as composites, are varied by the fiber extraction process [22]. Several methods have been studied to extract natural fibers, for example, mechanical decortications [23], water retting [22,24], dew retting [25], enzyme retting [26], etc. However, alkali (chemical) treatment was used in this experiment due to some advantages of this method over others. One of the main benefits of alkali treatment is that it removes impurities such as oil, wax, fat, pectin, hemicellulose and lignin from the fiber surface and increases the roughness of the fiber surface, thus resulting in better fiber matrix adhesion and consequently higher strength and toughness of the composites [27]. In our previous studies, a significant increase in the tensile strength of natural fiber nonwovens and composite materials after alkali treatment was reported [28,29]. Furthermore, the fibers obtained by the alkali extraction process exhibit higher strength and higher cellulose content than other processes [26,30,31]. Gamma radiation is a powerful radiation which can reorganize the polymer structure and create a more crystallized structure of the material. Gamma radiation has been successfully applied in several studies to increase the mechanical properties of NFPCs [32,33,34,35]. In the current study, five different doses of gamma radiation, e.g., 100–500 krd were applied to the composites. Water repellent (WR) chemical cross-links with the hydroxyl group of cellulose on the fiber surface and creates a thin layer which blocks the water molecules from penetrating inside. The WR chemical is usually used for a hydrophobic finish in textiles. However, the application of water repellent in NFPCs is a new approach. In this study, different concentrations of water repellent, e.g., 5–10% were applied on the nonwoven surface before reinforcing them. 

Therefore, the experimental works includes extraction of fibers from water hyacinth petiole and sugarcane bagasse by chemical process, fabrication of nonwovens by wet-laid web formation and development of composites by reinforcing the prepared nonwovens on two polymer matrixes, e.g., unsaturated polyester resin and epoxy resin. Two types of surface treatments were applied, such as water repellent pretreatment in nonwovens and gamma radiation in composites. The samples were analyzed by SEM to investigate the fiber surface and tensile fracture, by EDX to determine the elemental compositions, by FTIR to identify the chemical functional groups and by XRD to determine the crystalline and amorphous profiles of the materials. Water absorbency and tensile properties, e.g., tensile strength, elongation at break percentage and tensile modulus were investigated for all of the composite samples before and after treatments. 

## 2. Materials and Methods

### 2.1. Materials

**Figure 1:**
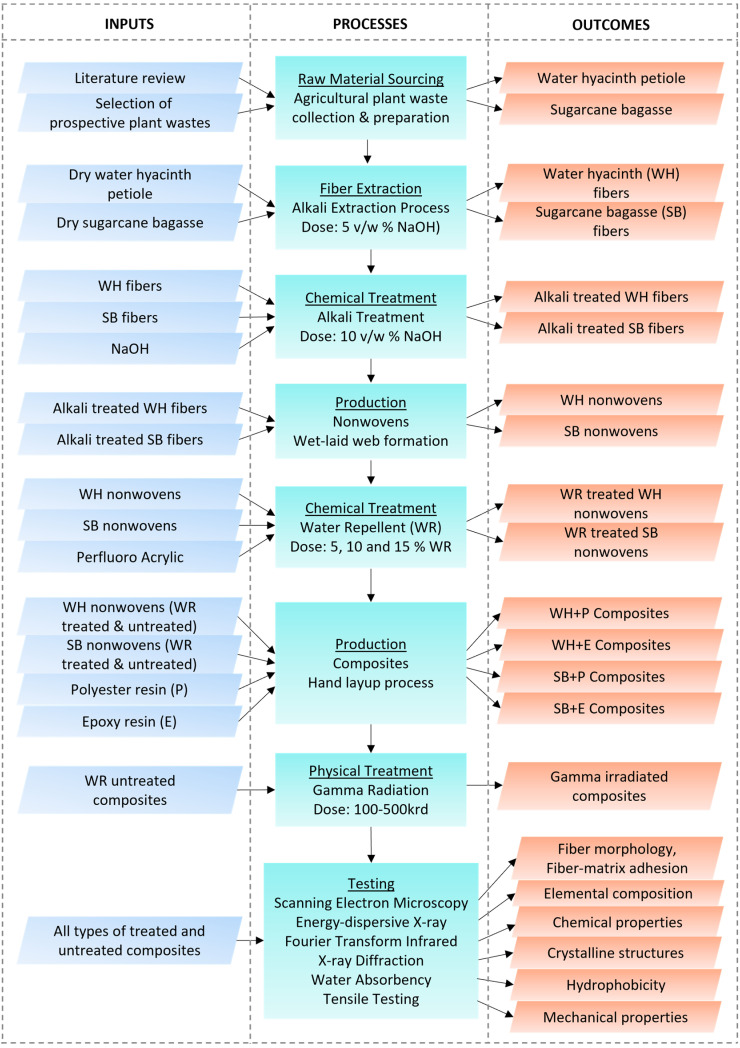
Experimental process scheme.

Water hyacinth (*Eichhornia crassipes*) plants were collected from a pond in Sirajganj, Bangladesh. The sugarcane bagasse (*Saccharum officinarum*) was collected from a garbage container of a juice producer after extracting the juice. Polyester and epoxy resin, hardener HY-951 and catalyst methylethylketone peroxide (MEKP) were bought from the manufacturer Nord Composites, Condé-Folie, France. Caustic soda (NaOH) and water-repellent chemical (perfluoro acrylic) were purchased from the manufacturer Archroma International Ltd., Mölndal, Sweden. High purity, laboratory-grade chemicals were used in this experiment. A process scheme is presented in Figure 1 to get an overview of the complete study. 

### 2.2. Methods 

#### 2.2.1. Fiber Extraction and Alkali Treatment 

For the extraction of WH fibers, petioles of WH plants were cut with a knife to separate them from the roots and leaves. The WH petioles were then exposed to the sun for a month, drying them out entirely and making them hard. On the other hand, after the juice was extracted using a mechanical squeeze roller, sugarcane bagasse (SB) was separated. After that, they were exposed to the sun for roughly two weeks to finish drying. Cut into tiny lengths of 5 cm, the dried WH petiole and sugarcane bagasse were stored separately. These little pieces were heated at 90 °C for 30 min until they softened in a solution of 5 (*v*/*w*)% NaOH in order to extract the primary fibers. To eliminate as much of the remaining NaOH and undesirable dusts as feasible, they were filtered from the caustic solution and rinsed under water. To obtain the fibers, they were then thoroughly dried for three hours in the oven. 

After being extracted, the raw fibers were treated with 10 percent (*v*/*w*) NaOH in the recommended concentration. For 24 h, fibers were submerged in a NaOH solution in a beaker. They were then taken out of the solution and given a thorough rinsing. After that, the fibers were left in the sun to finish drying. Alkali treatment is known to eliminate lingering contaminants from the fiber surface and subsequently improve mechanical strength. However, a higher concentration of alkali can damage the polymeric structure. Therefore, it is necessary to maintain the optimal dose. In this case, the optimal dose is 10% NaOH (*v*/*w*), which was determined from our earlier experiments. 

#### 2.2.2. Nonwoven Development 

**Figure 2:**
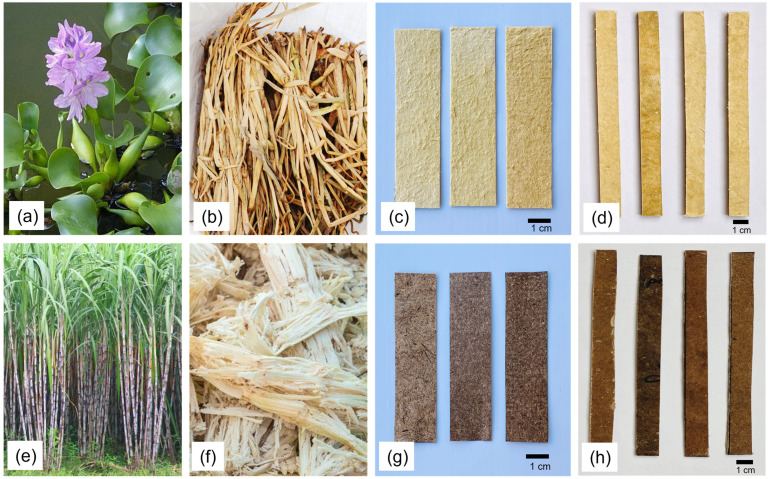
(**a**) Water hyacinth plant, (**b**) dry water hyacinth petiole, (**c**) water hyacinth nonwovens, (**d)** water hyacinth nonwoven reinforced composite specimens for tensile test, (**e**) sugarcane plant, (**f**) dry sugarcane bagasse, (**g**) sugarcane bagasse nonwovens and (**h**) sugarcane bagasse nonwoven reinforced composite specimens for tensile test.

In order to create a fiber pulp, the prepared dry fibers were blended with the water. A mesh was used to separate the fiber pulp and rinse them thoroughly to remove extra NaOH and other contaminations. A further 50 g of this fiber pulp was added to the blender, along with water in a 1:50 pulp-to-water ratio. They were combined until they were homogeneous and supple. After that, the slurry was put into a specifically designed perforated mold comprised of mesh and frame of hardwood. For a homogeneous distribution of the fibers inside the mold, the entire mold was submerged in water Table In order to drip water through the mesh, the mold was then positioned between two supports. After the water drips had stopped, an absorbent paper gently squeezed the formed pulp sheet or nonwoven to remove extra water. After that, it was removed from the mold and dried in the sun. If required, the nonwoven was strengthened using an electrical iron or a dead weight. Figure 2 displays the ready-to-use nonwovens. For WN and SN nonwovens, the average thickness was determined to be 0.8 mm and 1.0 mm, respectively.

#### 2.2.3. Application of Water Repellent 

A pad-dry-cure approach was used to apply a water repellent (WR) chemical to the ready nonwovens in order to increase the hydrophobicity of composites. Three different WR concentrations (5, 10, and 15% (*v*/*v*)) were applied to nonwovens, and the excess chemicals were padded off. They were dried and cured simultaneously for 30 min at a temperature of 160–180 °C. 

#### 2.2.4. Composite Fabrication 

A simple manual technique called hand-layup was used to fabricate the composites. The prepared WH and SB nonwovens were reinforced on two types of matrices, that is, unsaturated polyester resin (UPR) and epoxy resin to make four types of composites separately, which are designated as WH + E, WH + P, SB + E and SB + P. Two steel plates with the dimensions of 35 cm × 35 cm × 0.2 cm were used to create a mold. The plates were covered with a layer of Teflon paper (PTFE), which was used as a demolding paper to avoid sticking between the mold plates and the composites. For each composite, three layers of nonwovens were reinforced. Each layer of non-woven fabric was prepared with a size of 30 cm × 30 cm. A three-layer set was weighed together and on the basis of that weight, a matrix solution, i.e., mixture of resin and hardener, was prepared to make a fiber/matrix weight ratio of 30:70. Then, 2% of MEKP was used for UPR and 10% of HY951 was used for epoxy composites, respectively, as a hardener. At first, one quarter of the resin solution was poured and spread over the steel plate. A layer of nonwoven was then placed on that and pressed uniformly by a hand roller, so-that the resin can penetrate easily through the nonwovens. The same procedure was carried out after adding another quarter of the resin to the first layer of nonwoven. Following this procedure, 3 layers of nonwovens were stacked one after another. After applying the final quarter of the resin on the third layer of nonwoven, another steel plate was placed on top of that. For a 24-h curing period, some dead weights were attached to the complete arrangement. Dead weights were then taken out, and the formed composite was then taken apart from the steel plates. The composites were 2.5 ± 0.5 mm in thickness on average. The prepared composite samples were designated with a code which are presented in Table 1. 

#### 2.2.5. Gamma Radiation 

To improve the mechanical properties of the composites they were irradiated with different doses of gamma radiation. At the Institute of Radiation and Polymer Technology, BAEC, Savar, Dhaka, Bangladesh, a Co-60 gamma irradiator with a remote-control electromechanical system and a 65 Kci capacity was employed (gamma beam 650, model 11R). Gamma beams were loaded from the source GBS-98 which is equivalent to 36 double-encapsulated capsules. Samples were exposed to radiation at various dosages, such as 100, 200, 300, 400 and 500 krd.

#### 2.2.6. Scanning Electron Microscopy (SEM)

The microstructure of the fiber surface and tensile failure of the nonwovens under various surface treatments were examined using a Quanta 200 FEG field emission scanning electron microscope (FESEM) at the Institute of Materials Science (IMS), Kaunas University of Technology (KTU), Kaunas, Lithuania. Samples of 1 cm × 1 cm were prepared and placed on the sample holder under the FESEM. For taking the images, 10–20 kV accelerating voltage and 10–130 Pa low vacuum were applied. 

#### 2.2.7. Energy Dispersive X-ray (EDX) 

The elemental composition of the composite samples was evaluated using a Bruker (X-ray 4030), Bremen, Germany, X-ray energy dispersion spectrometer at IMS, KTU, Kaunas. By identifying chemical elements from B to Am in a chosen spot (1 m^3^ volume), the spectrometer provides quantitative and qualitative evaluation of the composition of a sample. An energy resolution of 133 eV (at Mn K line) is guaranteed by a modern 30 cm^2^ temperature-controlled (Peltier element) X-ray silicon shift spectrometer detector, which also has a very high X-ray photon detection speed (100,000 impulses per second).

#### 2.2.8. Fourier Transform Infrared (FTIR)

The chemical functional groups of the fibers and composites were investigated by a VERTEX 70 (from Bruker, Bremen, Germany) FTIR with attenuated total reflectance (ATR) at IMS, KTU, Kaunas. Changes in functional groups before and after surface treatments were also evaluated with FTIR. The spectrometer is able to determine the spectra with a range of 500–4000 cm^−1^ at a resolution of 1 cm^−1^.

#### 2.2.9. X-ray Diffraction (XRD) 

A D8 Discover X-ray diffractometer (Bruker AXS GmbH) was used to determine the crystal structure of fibers and composites. The diffractometer utilized a Cu K (λ = 1.5418) radiation source, parallel beam geometry, and a 60 mm Göbel mirror while operating at 40 kV and 40 mA. A fast-counting LynxEye detector with a 2.475° opening angle and 6 mm slit opening was used to record the diffraction pattern. Peak intensities were scanned with a 0.02° step size throughout a range of 10 to 70° (coupled 2– scans). XRD data were analyzed using the software DIFFRAC.EVA version V3.0 (Bruker). On the X-ray beam and detector sides, 0.6 mm divergence and 4 mm anti-scatter slits were utilized to reduce the effects of air scattering and Compton scattering, respectively. To represent scattering from the sample rather than environmental effects, the two-point linear background was subtracted from the fitted XRD data. The ratio between the area belonging to the crystalline phase and the overall area of the XRD pattern beneath the curve was used to determine the crystallinity index percentage (CI%). For all samples, the same range of 10–50° was used to calculate the degree of crystallinity. For all samples, the same range 10–50° 2*θ* was used to calculate the CI%. 

#### 2.2.10. Water Absorbency Test

The standard ASTM D570-98 was followed for measuring the water absorbency of the composites. Samples were conditioned at 50 °C for 24 h in an oven and cooled in a desiccator. Before putting the samples in water, they were weighed, which was considered as dry weight. For measuring the weight of samples in different duration of the water immersion, they were taken out from the water for a period of maximum 2 min in every 12 h up to 72 h. After 72 h all the samples were found stable in weight thus, the immersion was continued until 72 h. Before measuring the weight, they were wiped with dry tissue paper. The following Equation (1) was used to calculate the water absorbency percentage.

(1) $$\mathrm{Water}\;\mathrm{Absorbency}\;\left(\%\right)=\frac{W_{w}-W_{d}}{W_{d}}\times 100$$

where *W_w_* is the weight water immersion and *W_d_* is dry weight. 

#### 2.2.11. Tensile Test 

A universal testing machine from ZwickRoel was used at Faculty of Mechanical Engineering and Design, Kaunas University of Technology, Kaunas, Lithuania to perform the mechanical test. The test was conducted in accordance with ASTM D638-14 standard. The examples were made with dimensions of 165 × 13 mm in length. The machine was programmed with a tensile displacement rate of 10 mm/min and a gauge length of 50 mm. The following Equations (2)–(4) were used to determine the tensile strength (TS), elongation at break (Eb%), and tensile modulus (E), accordingly.

(2) $$\mathrm{TS}=\frac{F_{max}}{A}$$

where *F_max_* = maximum force before break and *A* = cross-sectional area of the sample.

(3) $$\mathrm{Eb}\%=\frac{L_{f}-L_{i}}{L_{i}}\times 100$$

where *L_i_* = initial length of the sample and *L_f_* = length of the sample at breaking point.

(4) $$E=\frac{d\sigma }{d\varepsilon }$$

where *dσ* = yield point stress and *dε* = yield point strain. 

## 3. Results and Discussion 

### 3.1. Scanning Electron Micrographs Analysis 

**Figure 3:**
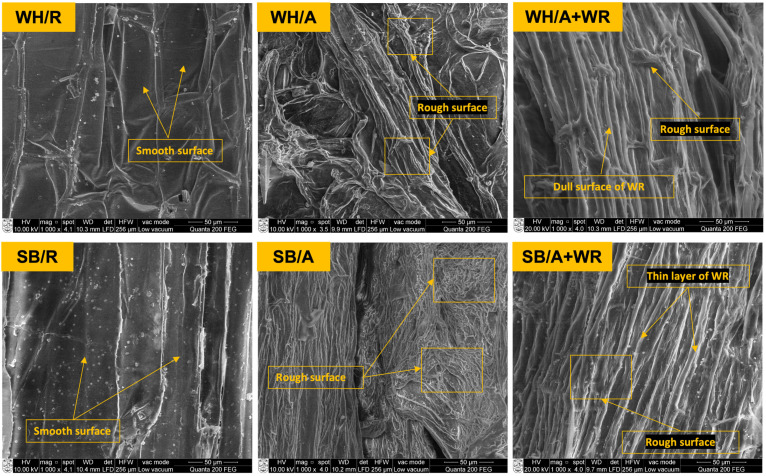
SEM micrographs of untreated (WH/R and SB/R), alkali-treated (WH/A and SB/A) and water-repellent-treated (WH/A + WR and SB/A + WR) water hyacinth and sugarcane bagasse fiber surface.

SEM micrographs of the raw untreated fiber surface, the alkali-treated fiber surface, and the water-repellent-treated fiber surface are presented in Figure 3. The raw water hyacinth (WH/R) and sugarcane bagasse (SB/R) fibers surface were mostly found to have a smooth surface due to the existence of natural impurities such as fats, wax, pectin etc., and some mineral impurities such as sodium, calcium, magnesium etc., on the surface of the fibers. However, after alkali treatment, the extracted fibers (WH/A and SB/A) were found to be rougher on their surface due to the elimination of the impurities mentioned above from the fiber surface. 

This surface roughness has very positive consequences that include not only better interlocking among the fibers when nonwovens were made, but also better adhesion and strong bonding between the fibers and matrix when composites were prepared from them. Due to this fact, all fibers used in this study were treated with 10% (*v*/*w*) NaOH (which was found to be an optimal dose for these fibers from another study) before using them to make the nonwovens and subsequently the composites. 

Water repellent (WR) chemical was applied to reduce the water absorbency of the composites. The dull surface is appeared on the WR treated fibers is caused by a thin layer after WR application. Water molecules are often prevented from entering the fibers by the WR polymers, which typically cross-link with the -OH groups and block them. The micrographs show enough surface roughness for interlocking among the fibers, which is essential in the nonwoven making process. However, fiber–matrix adhesion may be affected after WR treatment because of the interruption of direct cross-linking between the cellulose and matrix polymers at higher concentration. 

### 3.2. Energy Dispersive X-ray (EDX) 

**Figure 4:**
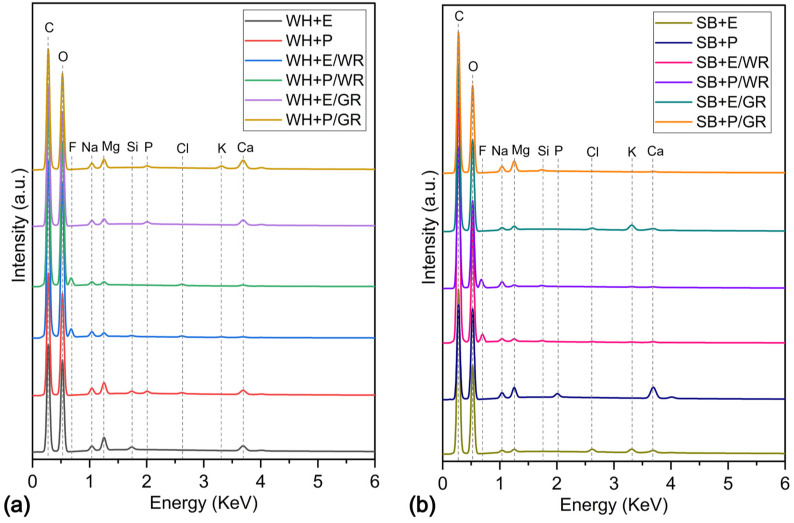
(**a**) EDX spectrum of standard (WH + E, WH + P), water-repellent-treated (WH + E/WR, WH + P/WR) and gamma-irradiated (WH + E/GR, WH + P/GR) water hyacinth composites, and (**b**) EDX spectrum of standard (SB + E, SB + P), alkali-treated (SB + E/WR, SB + P/WR) and gamma-irradiated (SB + E/GR, SB + P/GR) sugarcane bagasse composites.

Table 2 displays the elemental composition of the standard, WR (10 *v*/*v*%) treated and gamma irradiated (200 krd) WH and SB composites ascertained by the X-ray energy dispersion spectrometer expressed by the percentage of atoms (at.%). EDX spectrums of the similar sets of the specimens are described in Figure 4. It is important to keep in mind that the intensity (cps/eV) depends on not only the elemental atomic percentage but also atomic number, absorption, fluorescence, etc., and they vary from element to element. Particularly, if the materials constitute a combination of light and heavy elements, the light elements such as O, C and F may not correspond with their actual atom/weight percentages. Thus, the spectrum mentioned in Figure 4 is only for the detection of the elements, not for their amounts. In general, two main elements C and O were detected for all the samples, which is about 95–99% (together with C and O) of the total amount of atoms of the material. Although most of the natural and mineral impurities were eliminated from the fiber surface by the application of NaOH, a very negligible amount of minerals still exists in the composites for all the specimens commonly. 

In the WH + E and WH + P composites, O was found to be the highest at.% which was 56.84 and 56.50 respectively. Another main element, C, was detected at 40.11 and 40.48% respectively for WH + E and WH + E. After WR treatment, the amount of C was noticeably increased by 7.18% and 10% for WH + E/WR and WH + P/WR composites, respectively. This is due to the cross-linking between WR polymers and the -OH groups of cellulosic polymers on the amorphous regions of the fibers. Therefore, number of O atoms was significantly reduced by 11.45 and 12.83% for the WH + E/WR and WH + P/WR composites, respectively. The presence of a notable amount of new element, F (4.87% for WH + E/WR and 4.50% for WH + P/WR), proves the appearance of the fluorine-based WR chemical (perfluoroalkyl acrylic) on the fiber surface. There were no significant changes of elemental composition in gamma irradiated WH composites compared to standard WH composites. 

The standard SB composites also contain C and O as main elements; however, the amount of C was found to be lower, and O was found to be higher in SB + P compared to SB + E. One possible reason for this is the lower polymeric cross-linking between the fiber and the polyester resin in SB + P composites. However, after gamma radiation, the amount of C increased significantly by 7.26% for SB + P/GR composites due to higher fiber–matrix adhesion. Similar changes in the elemental compositions were observed for SB composites as WH composites after WR treatments as the WH composites. That is, a noticeable increase in C, a decrease in O and the appearance of a new element, F. These changes were due to the application of WR chemicals. As described above, WR perfluoro acrylic cross-links with OH groups of cellulosic polymers on the amorphous regions of the fibers.

### 3.3. Fourier Transform Infrared (FTIR) Analysis

**Figure 5:**
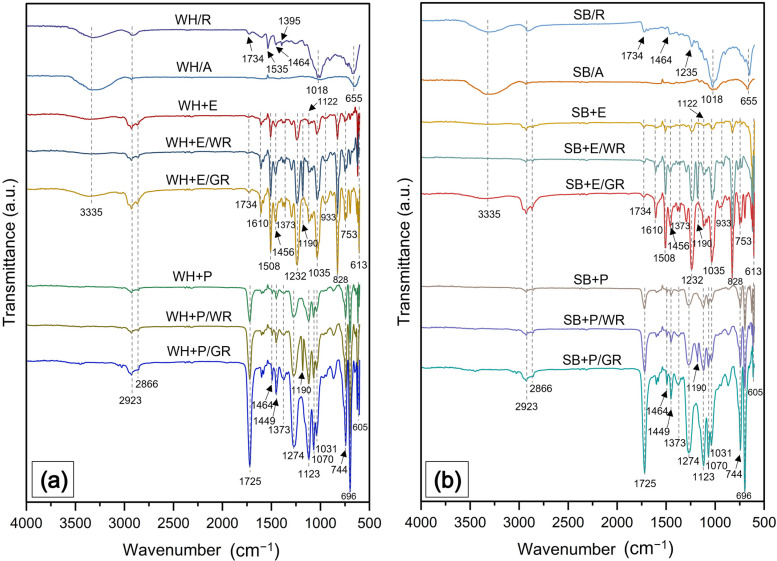
(**a**) FTIR spectrum of standard (WH + E, WH + P), water-repellent-treated (WH + E/WR, WH + P/WR) and gamma-irradiated (WH + E/GR, WH + P/GR) water hyacinth composites, and (**b**) EDX spectrum of standard (SB + E, SB + P), alkali-treated (SB + E/WR, SB + P/WR) and gamma-irradiated (SB + E/GR, SB + P/GR) sugarcane bagasse composites.

FTIR spectra of raw fibers, alkali-treated fibers, standard composites, WR-treated composites, and gamma-irradiated water hyacinth composites are shown in Figure 5a and sugarcane bagasse in Figure 5b. Both the raw fibers, i.e., WH/R and SB/R, exhibited mostly a similar type of spectrum. Some common peaks were observed, such as at 3335, 2923, 1734, 1464, 1018 and 655 cm^−1^, which are responsible for the OH group of cellulose and hemicellulose, C-H bending of hemicellulose, C=O bending of hemicellulose, -CH stretching of lignin, ester C-O and alkane C=C stretching, respectively. The peak at 1535 cm^−1^ detected in WH/R corresponds to the C=O bending of hemicellulose, and the peak at 1235 cm^−1^ detected in SB/R assigned to the C-O-C group proves the presence of lignin. Therefore, both raw fibers contained not only their main constituent cellulose but also some impurities such as hemicellulose, lignin and other natural impurities such as fat, wax, pectin, etc. However, the alkali-treated fibers i.e., WH/A and SB/A most of the peaks that are responsible for the impurities disappeared or reduced significantly. These prepared fibers were used to make the nonwovens and subsequently the composites. 

The spectrum was different for epoxy matrix composites and polyester matrix composites. Standard epoxy composites for WH and SB reinforcement, i.e., WH + E and SB + E showed similar spectra. The functional groups of epoxy resin were confirmed by the peaks at 1610 and 1508 cm^−1^ associated with C=C vibration of the benzene ring of epoxy [36], the peak at 1456 cm^−1^ corresponding to the H-C-H groups [37] and peaks at 1232 and 1035 cm^−1^ assigned to the C-O-C stretching of ether linkage [38]. The lower intensity at 933 cm^−1^ reveals that the epoxy resin was completely cured [36]. Aromatic ring out-of-plane stretching and substituted aromatic ring stretch of epoxy were observed at peaks 828 cm^−1^ and 753 cm^−1^ respectively [39]. On the other hand, the significant reduction of the 3335 cm^−1^ peaks in the composites determines the crosslinking between the -OH group of the cellulose and the epoxy resin. The peaks observed at 2923 cm^−1^ and 2866 cm^−1^ correspond to the C-H stretching of cellulose and epoxy polymers, which revealed the interaction between the fiber and the matrix. A small peak at 1734 cm^−1^ is assigned to the C=O of hemicellulose, at 1373 cm^−1^ is assigned to the C-H symmetrical deformation of cellulose and at 1122 cm^−1^ is assigned to C-O-C asymmetrical stretching of cellulose and hemicellulose [40]. These peaks also determine the fiber–matrix cross-linking. Water-repellent-treated epoxy composites, i.e., WH + E/WR and SB + E/WR showed similar spectra. An additional peak at 1190 cm^−1^ was observed for the composite that belongs to the stretching of -CF2, which reveals the presence of fluorine-based WR on the composites and their interaction with the matrix. The overall intensity was increased in WR-treated epoxy composites compared to that in standard epoxy composite, which may be due to polyaddition among the cellulose, WR and epoxy. Gamma-irradiated epoxy composites exhibited similar spectra as standard composites with significantly higher intensity, especially at the wavelengths 3335, 2923, 1373 and 1122 cm^−1^, which are responsible for functional groups of the cellulose, which determines the better fiber–matrix adhesion. The increase of peak intensity shown by the epoxy polymers e.g., 1610, 1456, 1232, 1035, 828 and 753 cm^−1^ also ensure the improved capacity of functional groups to cross-link with the fiber. 

The standard composite made with unsaturated polyester resin i.e., WH + P and SB + P showed several different peaks compared to epoxy composites. The main peaks that are responsible for the functional group of polyester resin appeared at 1725, 1449, 1274, 1123, 1070 and 744 cm^−1^, corresponding to the carbonyl stretching C=O from the ester linkage, aromatic ring of phenyl group, twisting vibration of -CH2 group, aliphatic ether/ester -COO-, unsaturated in-plane deformation and unsaturated aromatic out-of-plane deformation, respectively [41,42,43]. The disappearance of the peak at 3335 cm^−1^ reveals the cross-linking between the -OH group of cellulose and the polyester resin. Moreover, the peaks that are responsible for the cellulose polymers observed at 1464, 1373 and 1031 cm^−1^ indicate the fiber–matrix interaction. Water-repellent-treated polyester composites, i.e., WH + P/WR and SB + P/WR exhibited an additional peak at 1190 cm^−1^ corresponding to the bending of -CF2 indicating the application of fluorine-based WR in both the WH and SB fibers. A notable increase in peak intensity was also evident after WR treatment. However, the composites after gamma radiation, i.e., WH + P/GR and SB + P/GR exhibited significantly higher peak intensity than the standard composites, particularly at 2923 and 2866 cm^−1^. This corresponds to the CH stretching of cellulose and polyester resin and reveals better cross-linking and adhesion between the fiber and the matrix.

### 3.4. X-ray Diffraction (XRD) Analysis

**Figure 6:**
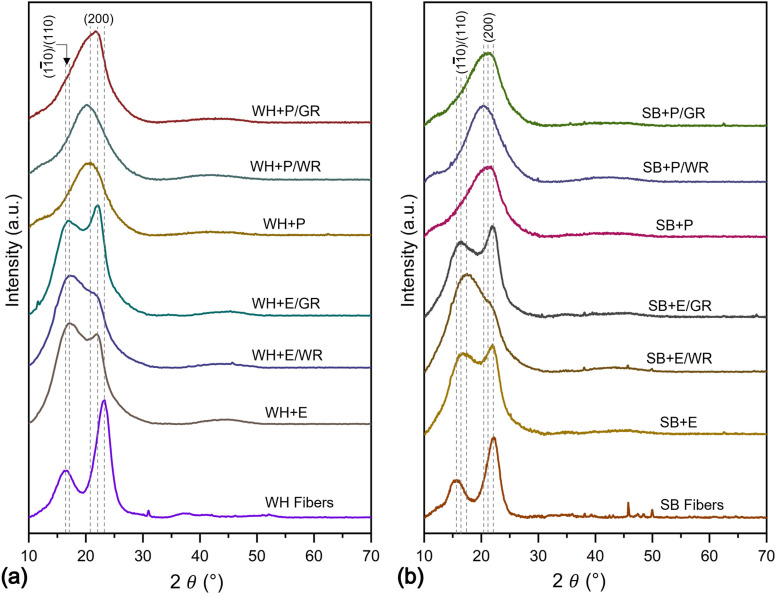
(**a**) XRD spectrum of standard (WH + E, WH + P), water-repellent-treated (WH + E/WR, WH + P/WR) and gamma-irradiated (WH + E/GR, WH + P/GR) water hyacinth composites, and (**b**) EDX spectrum of standard (SB + E, SB + P), alkali-treated (SB + E/WR, SB + P/WR) and gamma-irradiated (SB + E/GR, SB + P/GR) sugarcane bagasse composites.

Figure 6 demonstrates the XRD spectrum of the WH and SB fibers and their composites under different surface treatments. The crystallinity index percentage (CI%) of all samples is presented in Table 3. Two major peaks were observed at 2θ 16.3° and 23.15° for WH fibers; and 15.5° and 22.1° for SB fibers which correspond to the crystallographic planes of (1$\bar{1}$0)/(110) (overlapped) and (200), respectively. The achieved results correlate with the standard crystallographic profiles of cellulose [44,45,46]. The intensity of both main peaks was higher for WH fibers than for SB fibers. Therefore, the percentage of CI of WH fibers was found to be higher (66.6%) than that of SB fibers (59.8%). 

The XRD spectrums of epoxy and polyester composites exhibited differently for both WH and SB reinforcements. Standard epoxy composites also showed two main peaks, for example, 17.2 and 22.1 for WH + E and 16.7 and 22.1 for SB + E composites, respectively. Thus, the angles were shifted slightly to the left or right for both composites, which determines the interaction of epoxy resin with the fibers. The intensity at the position of ~16–17° was remarkably increased for both the composites. However, the intensity at ~22° was reduced for WH + E and increased slightly for the SB + E composites. This scenario reveals the presence of cellulose, epoxy, and their interactions. The CI% of WH + E composite was reduced slightly to 66.0 from CI% of the fibers but increased significantly in case of SB + E composites to 64.5. This may be due to the addition of crystalline epoxy polymers with the fibers. However, the WH + E composite is still showing a higher CI% than SB + E due to the noticeably higher CI% of WH fibers. The WR-treated epoxy composites showed different patterns of spectra compared to the standard epoxy composites. Peak intensity at ~22° was significantly reduced for both WH + E/WR and SB + P/WR composites. Therefore, the CI% of both composites were reduced compared to that of the standard composites. 

On the other hand, the peak intensity at ~16–17° was increased for SB + E/WR and decreased for WH + E/WR composites. Thus, the decline of CI% for SB + E/WR is less than that of the WH + E/WR. The overall reduction in CI% for the WR treated composites is due to the interruption of fiber–matrix cross-linking by the thin coating of WR chemicals on the fiber surface. However, the gamma-irradiated composites showed a higher percentage of CI than the standard compounds of 66.6 to 67.2 for WH + E/GR and of 59.8 to 65.3 for the SB + E/GR composites. A higher intensity at the two main peaks, i.e., ~16° and ~22° also evidently increase the CI%. This may be due to the fact that gamma radiation creates reactive sites for both fiber and matrix that help to cross-link each other strongly in their amorphous regions and increase the crystallinity of the materials.

The composites made with polyester resin matrix showed a spectrum noticeably different from those of their respective fibers. Two major changes were observed from the fibers to the composites. For example, the disappearance of the peak at ~16–17° and the shifting of the peak at 22–23°, which was common for both the WH and SB polyester composites. The only main peak was detected at 20.8° for WH + P and at 21.2° for SB + P composites which determines to the incorporation and domination of polyester resin over the fibers [47]. The CI was decreased notably by 19% and 16% for WH + P and SB + P composites from the WH and SB fibers, respectively. After WR treatment the peaks were shifted to the left to 19.9 and 20.3 for WH + P/WR and SB + P/WR composites respectively. This may be due to the incorporation of WR chemicals on the fiber surface. The spectrum of gamma irradiated composites showed a shifting to the right for both the WH + P/GR and SB + P/GR composites than the standard polyester composites. Gamma radiation can rearrange the polymeric structure of the fiber and matrix by creating more adhesion among them. Thus, the CI was improved significantly by 8% and 13% for WH + P/GR and SB + P/GR composites, respectively.

### 3.5. Water Absorbency Analysis

WH + E exhibited the lowest water uptake of 11.21% and SB + P exhibited the highest water uptake of 14.23% after 72 h of continuous immersion in water. Epoxy composites showed a lower water absorbency than polyester composites. For example, WH + E showed 15.3% and SB + E showed 10.9% lower water uptake (%) than the WH + P and SB + P composites, respectively. This scenario reveals the higher water-resisting capacity of epoxy resin and better fiber–epoxy adhesion compared to polyester composites. It is also evident from the XRD results that the CI% of epoxy composite was significantly higher than that of the polyester composites. Thus, the higher amorphous area of polyester composites allows more water to penetrate the materials. On the other hand, WH composites demonstrated lower water uptake (%) than SB composites. For example, WH + E showed 11.1% and WH + P showed 6.9% lower water uptake (%) than SB + E and SB + P composites respectively. This episode determines the better fiber–matrix adhesion for WH composites and the lower water absorbency of WH fibers than the SB composites and SB fibers, respectively. The CI% also correlate with this phenomenon as the WH composites found slightly higher CI% than the SB composites. 

**Figure 7:**
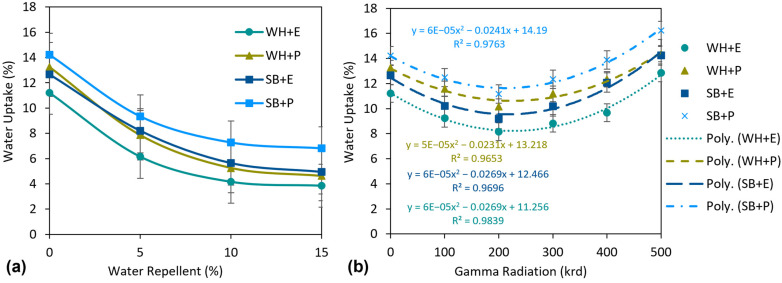
(**a**) Effect of water-repellent treatment and (**b**) effect of gamma radiation on the water uptake (%) of the composites.

The application of WR chemical on the nonwoven surface dramatically affects the water absorbency of the composites. The results are demonstrated in Figure 7a. The water uptake percentage was decreased by 45.1%, 40.7%, 35.2% and 34.3% for WH + E, WH + P, SB + E and SB + P composites, respectively, at the WR dose of 5 (*v*/*v*)% compared to untreated composites. Water uptake (%) continued to decrease at a dose of 10% WR. For example, water uptake (%) was reduced by 62.9% for WH + E, 60.3% for WH + P, 55.4% for SB + E and 48.8% for SB + P composites than untreated composites. For the further increase in WR dose at 15%, the water uptake (%) was still decreasing, however, the declination rate was very low compared to the rate at 5% and 10%. For example, only 2.8, 4.8, 5.5 and 3.2% of decrement of water uptake (%) was found at a dose of 15% WR than the water uptake (%) at 10% WR. Therefore, it is worth applying a maximum 10% WR if the other properties do not deteriorate so much due to WR treatment. Cellulosic fibers are known for being highly water-absorbent materials. Thus, if they are used as a reinforcing material in a composite, there is always a chance of high water absorption of the composites. In this study, the WR chemical was applied to the surface of the reinforcing nonwovens. In the amorphous area of the fibers, a chemical water repellent forms cross-links with the cellulose polymer, forming a thin covering that prevents water from penetrating inside the fiber. Therefore, the overall hydrophobicity of the composites improved remarkably. 

The effect of gamma radiation on water uptake (%) is described in Figure 7b. The curves are prepared in second-order polynomial form because of alternative effects (positive and negative) on water absorbency under different doses of gamma irradiation. Initially, at a dose of 100 krd, the water uptake (%) was reduced by 17.6%, 12.5%, 19.4%, 12.3% and at a dose of 200 krd reduced by 27.2%, 23.3%, 27.5% and 21.5% for WH + E, WH + P, SB + E and SB + P composites, respectively, than unirradiated composites. However, the water uptake began to increase for further increasing of the gamma dose from 300 krd. The increment in water absorbency was continued for 400 krd and 500 krd of gamma radiation dose. For instance, an increment of 18.6%, 21.6%, 31.6% and 24.3% of water uptake (%) was observed at 400 krd gamma dose for WH + E, WH + P, SB + E, SB + P composites, respectively, than the lowest water uptake (%) at a dose of 200 krd. At 500 krd, the water uptake (%) was found to be even higher than the unirradiated composites. The decrease in water absorbency at a lower gamma radiation dose, i.e., 100–200 krd, is due to the improvement of the intermolecular linkage between the fiber and the matrix by reducing the interfacial resistance that restricts the water molecule from penetrate and disperse inside the materials. However, at a higher gamma radiation dose, for example 300–500 krd, the interfacial fiber–matrix adhesion was reduced because of the polymeric destruction of fiber and matrix by the intense gamma radiation dose. This increases the amorphous area of the composites and increase the water absorbency [47].

### 3.6. Mechanical Properties Analysis

Mechanical properties such as tensile strength, elongation at break and tensile modulus of all the composite materials, as well as the effects of water repellent and gamma radiation application on these tensile properties are described in this section. All parameters were statistically analyzed with a maximum coefficient of variation (CV) of 7.6%.

#### 3.6.1. Microscopic Analysis of Tensile Fracture 

**Figure 8:**
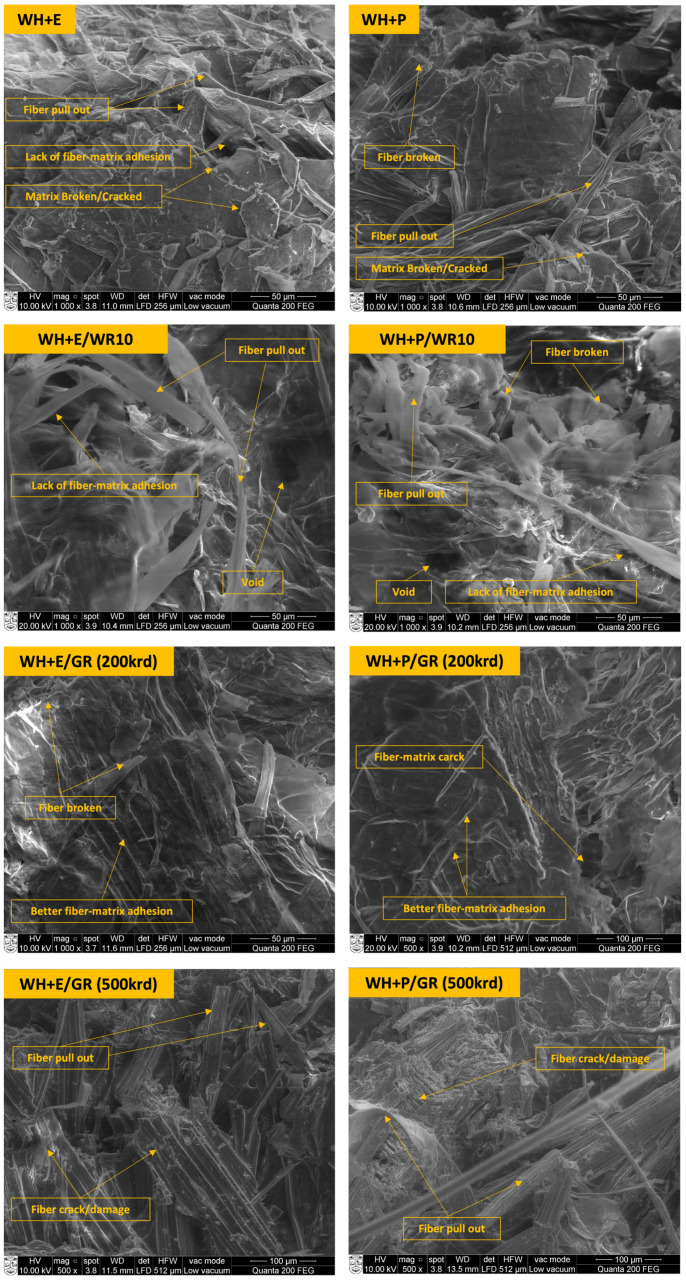
SEM micrographs on the tensile fractures of standard water hyacinth (WH) composites (WH + E and WH + P), water-repellent-treated WH composites (WH + E/WR and WH + P/WR) and gamma-irradiated WH composites (WH + E/GR and WH + P/WR).

Figure 8 presents the SEM micrographs of the tensile fracture of the untreated, WR-treated, and gamma-irradiated water hyacinth (WH) composite samples. Very similar types of micrographs were observed for sugarcane bagasse (SB) composites; therefore, it is worth analyzing only the micrographs of WH composites. From the tensile fracture of untreated WH-epoxy (WH + E) composites, it was found that some fibers were pulled out of the matrix due to the lack of fiber–matrix adhesion. In addition, some small gaps were found between the fiber and the epoxy matrix. Other than that, the fibers and matrix were cracked or completely broken in the loading position. Similar types of fiber pullouts, broken fibers, cracked and broken matrix were found in WH + P composites. More fiber pullouts were observed in the tensile fracture of both the WR treated (10 *v*/*v*%) composites WH + E/WR and WH + P/WR. Some gaps and voids between the fibers and the matrix were also found, indicating a significant lack of fiber–matrix adhesion. This is because, at a higher concentration of WR, most of the water-attracting -OH groups were blocked by the WR polymers, creating an interruption between the cross-linking of cellulose and matrix that leads to low adhesion between them and consequently lower mechanical properties of the composites. 

However, better fiber–matrix adhesion was found for the gamma irradiated composite samples. Tensile-fracture micrographs of WH + E/GR and WH + P/GR (at a gamma radiation dose of 200 krd) show a very low number of fiber pullouts. Rather, the fibers and matrix were broken in a regular way. The regular fiber orientation, homogeneous fiber distribution and strong bond between the fiber and the matrix were visible in the micrographs. These findings determine a very good fiber–matrix adhesion which can lead to a mechanical property better than that of nonirradiated composites. This is because of the penetration of strong ionizing gamma radiation into the composite materials, which influences the polymeric structure of the materials by producing reactive sites and subsequently enhanced matrix and fiber adhesion and cross-linking. An opposite scenario was found in the micrographs of WH + E/GR and WH + P/GR at a higher gamma radiation dose of 500 krd. Fiber and matrix were already damaged severely before applying the tensile load for both the composites samples, which is evident from micrographs. A scattered and irregular breaking of fiber and matrix occurred under the tensile load. In addition, a number of fibers were pulled out from the matrix. These characteristics of tensile failure determine weak fiber–matrix adhesion of the composites. This is because, at a higher dose of gamma radiation, the main polymeric chains were broken down rather than crosslinking; this phenomenon is also known as chain scission. 

#### 3.6.2. Tensile Strength 

**Figure 9:**
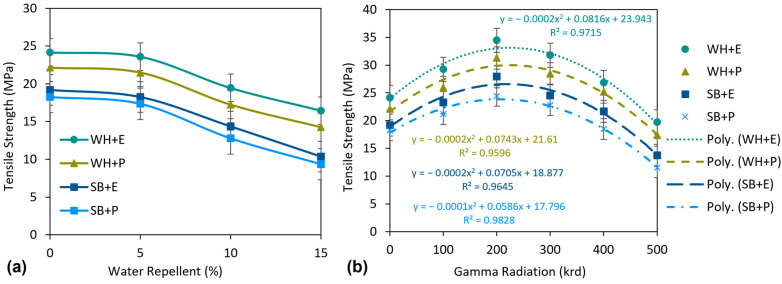
(**a**) Effect of water-repellent treatment and (**b**) effect of gamma radiation on tensile strength of the composites.

Figure 9 shows the tensile strength (TS) of the composites. Among the standard composites, WH + E exhibited the highest TS of 24.16 MPa and SB + P exhibited the lowest TS of 18.25 MPa. WH + E and WH + P showed 25.9% and 21.2% higher TS than the SB + E and SB + P composites, respectively. This significantly higher TS of WH composites than of SB composites is due to the higher strength of WH fibers/nonwovens and better adhesion of WH fibers with the matrixes than that of the SB fibers. XRD data also reveal a notably higher crystallinity of WH fibers than that of SB fibers, and the material with higher crystallinity expects a higher strength than the material with lower crystallinity, due to their well-organized and compact polymeric structure. On the other hand, WH + E and SB + E demonstrated 9.2% and 5.2% higher TS than WH + P and SB + P composite, respectively. This higher TS of epoxy composites is due to the higher strength of epoxy resin and its better interfacial bonding with the cellulosic fibers. A similar trend was reported in some previous investigations [48,49,50]. The effect of the water repellent on the TS of the composites is presented in Figure 9a. Although WR treatment greatly improved the hydrophobicity of the composites, they created a negative impact on the TS. The TS of the WR-treated composites was significantly reduced at higher concentrations of WR. For instance, TS was reduced by about 40% on an average when pretreated with 15% WR. However, at a lower concentration of WR treatment, i.e., 5% WR, the reduction in TS was not so high. The depletion was even less for WH + E and WH + P composites, only 2.4 and 2.9%, respectively, than for the untreated composites. Although there was a small reduction in TS at 5% WR, it is still worthwhile to apply this dose to achieve a significant improvement in the hydrophobicity of the composites.

This significant reduction of TS at higher concentrations of WR is due to the presence of a thin layer of WR on the surface of the fiber which covers the water-attracting -OH groups of fiber and resists the penetration of water molecules. On the other hand, this thin layer of WR may create an obstacle for better fiber–matrix adhesion, which results in lower mechanical properties of the composites. However, at lower concentrations of WR, the fiber–matrix adhesion was not affected so much.

Figure 9b represents the effects of gamma radiation on the TS of the composites. A second-order polynomial trendline is added for all the curves to show the increasing and decreasing trend of the TS with the increase of gamma radiation dose. The TS was increased remarkably with the increase of gamma does up to 200 krd. For example, TS increased by 42.7, 41.4, 45.5 and 33.9% for WH + E, WH + P, SB + E and SB + P composites, respectively, at a gamma dose of 200 krd than for untreated composites. However, TS started to decrease at a dose of 300 krd and continued this decrease to 500 krd. For instance, WH + E, WH + P, SB + E and SB + P composites showed 42.7, 44.4, 50.9 and 52.7% reduction of TS, respectively, at a dose of 500 krd compared to the TS at a dose of 200 krd.

The improvement in TS is due to modification or rearrangement of the polymeric structure of the composites at lower gamma radiation doses. In detail, gamma radiation is known as strong ionizing radiation, which can penetrate the polymeric structure of composites and create different types of reactive sites such as free radicals, ions and peroxides [33,34,35]. Therefore, these reactive species can cross-link each other and create a longer polymeric chain with larger molecules. This phenomenon is known as photo-crosslinking, which leads to better bonding between fiber and matrix, thus improving their adhesion properties and increasing the tensile strength [51]. However, at a higher dose of gamma radiation, the TS was dramatically decreased due to the occurrence of an opposite phenomenon which is called photodegradation. This leads to severely destructing the main polymeric chains of the material into small fragments and deteriorates the mechanical properties of the composites [33].

#### 3.6.3. Elongation at Break

**Figure 10:**
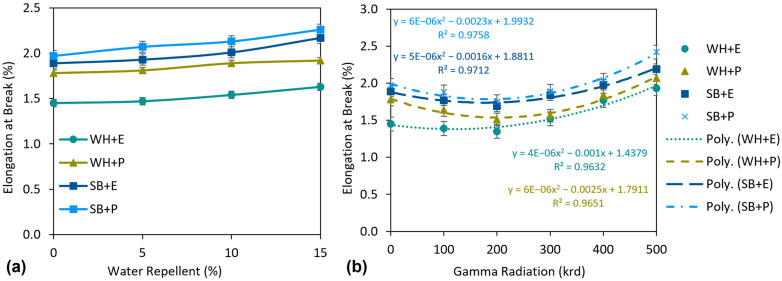
(**a**) Effect of water-repellent treatment and (**b**) effect of gamma radiation on elongation at break (%) of the composites.

The elongation properties of the composites are described in Figure 10. In general, all the composites showed relatively low elongation at break. The highest 1.97 Eb% was exhibited by SB + P composites and the lowest 1.45 Eb% was exhibited by WH + E. The WH composites showed lower Eb% than the SB composites and the polyester composites showed higher Eb% than the epoxy composites. Thus, the comparative results of Eb% were completely opposite to those of TS. This was expected because the lower the elongation of a material, the better the strength in general.

The effects of elongation properties on the WR treatment were not significant but still noticeable. The results are shown in Figure 10a. There was a gradual increase in Eb% with increasing WR concentrations. At lower concentration of WR, for example 5% WR, the increase in EB% was very low (about 2.6% on average) for all the composites. However, at a higher concentration such as 15%WR, Eb% increased by 12.5% considering the average value of all the composites. WR may cause interruption of fiber–matrix adhesion, as described above. Due to that, the crystallinity of the WR-treated composites may be reduced, which was also evident from the XRD analysis. Thus, more amorphous regions lead to an increase in the Eb% of the composites under tensile loads.

Figure 10b shows the influence of gamma radiation on the Eb% of the composites. Eb% decreased slightly with the increase of the gamma radiation dose to a certain dose of 200 krd. For example, Eb% was reduced by 12.6, 3.3, 8.9 and 8.0% for WH + E, WH + P, SB + E and SB + P composites, respectively, at a dose of 200 krd compared to untreated composites. However, the Eb% was increased for the further increasing of gamma dose, i.e., 300 krd and continued to increase up to 500 krd. An increase of 35.6, 19.2, 17.8 and 25.7% of Eb% was observed for the WH + E, WH + P, SB + E and SB + P composites respectively at a gamma dose of 500 krd compared to the Eb% at a dose of 200 krd, which is even higher than the untreated composites. Gamma radiation may rearrange the polymeric structure of the composites and form a more oriented polymeric structure of the materials by creating a strong bonding between the fiber and the matrix. Therefore, the composite has less space to extend under tensile load, which decreases the Eb% of the composites. On the other hand, the higher gamma dose, e.g., 300–500 krd, is responsible for the polymeric degradation of the materials. This leads to an irregular polymeric structure that creates more amorphous regions inside the material and consequently increases the Eb% of the composites.

#### 3.6.4. Tensile Modulus

**Figure 11:**
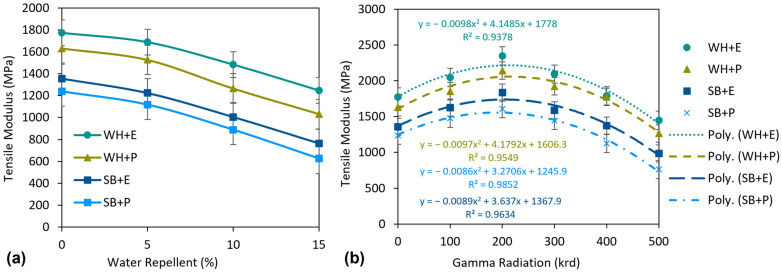
(**a**) Effect of water repellent treatment and (**b**) effect of gamma radiation on tensile modulus of the composites.

Figure 11 shows the tensile modulus (TM) of the standard composites and the effect of WR and gamma radiation on the TM of the standard composites. The composites, e.g., WH + E, WH + P, SB + E and SB + P, exhibited 1773, 1631, 1356 and 1238 MPa TM, respectively. WH composites, e.g., WH + E and SB + E showed 30.8% and 31.8% higher TM than SB composites, e.g., SB + E and SB + P, respectively. This higher TM of WH composites indicates not only the higher strength of WH fibers but also better fiber–matrix adhesion in comparison to SB composites. As discussed above, the WH composites contained more regular and crystalline polymeric structure, leading them to have a better TM than the SB composites.

There was an influence of WR treatments on the TM of the composites which is presented in Figure 11a. The TM of the composites decreased significantly with an increase in WR concentrations. For example, at 5% WR treatment, TM was reduced by 4.8, 6.4, 9.7 and 9.8% for WH + E, WH + P, SB + E and SB + P composites, respectively. At higher concentrations, i.e., 10–15% WR, the TM deteriorated at higher rates. For example, approximately 25% reduction of TM was observed at 10% WR and 43% reduction of TM was observed at 15% WR considering the average of all composites. The main reason is already described above. The WR chemical creates a thin coating on the nonwoven surface and this coating may interrupt the fiber–matrix bonding. This leads to lower TM of the composites than untreated composites. However, at lower concentrations, i.e., 5% WR, the TM was reduced less than 10%. Therefore this study recommends the application of WR with the maximum concentration of 5% to achieve a dramatic improvement in hydrophobicity of the composites.

The effect of gamma radiation on the TM is visible in Figure 11b. The TM of all composites increased to a gamma radiation dose of 200 krd. For example, TM of WH + E, WH + P, SB + E and SB + P composites was increased by 32.4, 31.3, 35.3 and 30.0%, respectively. However, the TM started to decrease after further increasing the gamma dose. An average (considering all types of composites) 11%, 24% and 45% decrement was observed at a dose of 300, 400 and 500 krd, respectively, compared to TM found at 200 krd. The significant increase in the TM of the composites by gamma radiation at lower doses is due to the same reasons as explained above for tensile strength. Gamma radiation interferes with the polymeric structure of the materials and creates reactive sites. These reactive sites from the fiber and matrix crosslink with each other and create a more crystalline and well-oriented polymeric structure. Therefore, the fiber–matrix adhesion was increased after gamma radiation and consequently the TM of the composite. However, at a higher gamma radiation dose, i.e., 300–500 krd, the TM of the composites started to decrease from the TM at 200 krd. This is because the higher gamma dose may damage the main polymeric chains on the composites, which is also known as chain scission. This destruction of the polymeric structures interferes with the fiber–matrix adhesion and reduced the TM of the composites. At 500 krd, severe destruction can occur and result in very low TM of the composites. The SEM image of the tensile fracture of the composites at 500 krd gamma dose also shows heavy destruction and lower fiber–matrix adhesion.

## 4. Conclusions

Newly developed nonwovens made from water hyacinth fibers and sugarcane bagasse were reinforced on polyester and epoxy matrix to make four types of composites. SEM micrographs showed a smoother surface of raw fibers, a rougher surface after alkali treatment and a thin coating after WR repellent treatment. The appearance of WR chemical was proved by EDX elemental composition and FTIR spectra. XRD spectra demonstrated higher crystallinity of gamma irradiated composites and lower crystallinity of WR treated composites than standard composites. SB composites (SB + E, SB + P) and polyester composites (WH + P, SB + P) showed higher water absorbency in comparison to WH composites (WH + E, WH + P) and epoxy composites (WH + E, SB + E), respectively. On the contrary, WH and epoxy composites exhibited higher mechanical properties (TS and TM) than SB and polyester composites. Water uptake (%) of the composites was markedly reduced (about 60% than untreated composites) by WR pretreatment up to 10% WR. However, after 10% WR, i.e., 15% WR, the reduction was not so significant. Gamma radiation also improved the hydrophobicity by about 25% (average) of the composites up to a dose of 200 krd. On the other hand, mechanical properties, e.g., TS and TM, deteriorated for the WR-treated composites in comparison to those of standard composites, but this reduction was less than 10% at a concentration of 5% WR, while about 40% reduction was found at a concentration of 15% WR. Therefore, to achieve a balance between mechanical properties and hydrophobicity of the composites, a maximum concentration of 5% WR is considered a recommended dose in this study. Gamma radiation was applied to improve the mechanical properties of the composites. Mechanical properties, for example, TS and TM were significantly improved by approximately 41% and 32%, respectively; and Eb% was reduced by approximately 11% at a dose of 200 krd than standard composites. However, TS, TM started to decrease and Eb% started to increase with further increase of dose at 300 krd. At 500 krd, the TS and TM of the composites decreased severely. Thus, 200 krd gamma radiation dose is considered as the optimum dose. This innovative development of nonwoven reinforced polymer composites by utilizing agricultural waste such as sugarcane bagasse and aquatic waste such as water hyacinth provided great potential for the application of packaging materials, bottles and containers, construction panels, interior furniture and shelves, automobile components, body of electronic devices and many more.

## Figures and Tables

**Table 1:** Sample designation.

Sample Description	Code
Standard water hyacinth nonwoven reinforced epoxy composite	WH + E
Standard water hyacinth nonwoven reinforced polyester composite	WH + P
Standard sugarcane bagasse nonwoven reinforced epoxy composite	SB + E
Standard sugarcane bagasse nonwoven reinforced polyester composite	SB + P
Water-repellent-treated water hyacinth nonwoven reinforced epoxy composite	WH + E/WR
Water-repellent-treated water hyacinth nonwoven reinforced polyester composite	WH + P/WR
Water-repellent-treated sugarcane bagasse nonwoven reinforced epoxy composite	SB + E/WR
Water-repellent-treated sugarcane bagasse nonwoven reinforced polyester composite	SB + P/WR
Gamma-irradiated water hyacinth nonwoven reinforced epoxy composite	WH + E/GR
Gamma-irradiated water hyacinth nonwoven reinforced polyester composite	WH + P/GR
Gamma-irradiated sugarcane bagasse nonwoven reinforced epoxy composite	SB + E/GR
Gamma-irradiated sugarcane bagasse nonwoven reinforced polyester composite	SB + P/GR

**Table 2:** Elemental composition of different WH and SB composite specimens before and after surface treatments.

Specimens ↓	Atom (%)									
Elements →	C	O	F	Na	Mg	Si	P	Cl	K	Ca
WH + E	40.11	56.84	-	0.69	1.23	0.69	-	-	-	0.44
WH + P	40.48	56.50	-	1.02	1.21	0.15	0.15	0.08	-	0.41
WH + E/WR	47.29	45.93	4.87	0.73	0.43	0.14	-	0.20	0.15	0.26
WH + P/WR	50.48	43.67	4.50	0.45	0.34	-	-	0.22	-	0.34
WH + E/GR	40.85	57.38	-	0.66	0.53	-	0.13	-	-	0.45
WH + P/GR	40.18	57.13	-	0.79	0.86	-	0.10	-	0.17	0.77
SB + E	47.09	51.84	-	0.29	0.22	-	-	0.15	0.22	0.19
SB + P	37.95	59.02	-	0.83	0.99	-	0.22	-	-	0.99
SB + E/WR	62.00	32.26	4.50	0.38	0.29	0.14	-	0.08	0.11	0.24
SB + P/WR	43.69	50.88	4.25	0.86	0.17	0.06	-	-	0.04	0.05
SB + E/GR	46.42	52.25	-	0.37	0.33	-	-	0.10	0.37	0.16
SB + P/GR	45.21	52.75	-	0.85	1.06	0.08	-	-	-	0.05

**Table 3:** Crystallinity Index (%) of WH and SB fiber and composite samples before and after surface treatments.

WH Samples	Crystallinity Index (%)	SB Samples	Crystallinity Index (%)
WH Fibers	66.6	SB Fibers	59.8
WH + E	66.0	SB + E	64.5
WH + E/WR	62.4	SB + E/WR	63.6
WH + E/GR	67.2	SB + E/GR	65.3
WH + P	53.7	SB + P	49.8
WH + P/WR	53.3	SB + P/WR	49.0
WH + P/GR	58.0	SB + P/GR	56.6

## Data Availability

Not applicable.
